# SHORT-TERM INTRAINDIVIDUAL DYNAMICS OF HEART RATE AND COGNITIVE FUNCTIONING IN OLDER ADULTS

**DOI:** 10.1093/geroni/igz038.3331

**Published:** 2019-11-08

**Authors:** Jonathan Rush, Tomiko Yoneda, Rebecca Venditelli, Jamie E Knight, Nathan A Lewis, Philippe Rast, Scott Hofer

**Affiliations:** 1 University of Victoria, Victoria, British Columbia, Canada; 2 University of California, Davis, Davis, California, United States; 3 University of Victoria, Victoria, B.C., United States

## Abstract

Short-term intraindividual variability in cognition can provide insights into the underlying processes that cannot be captured by only examining mean levels (MacDonald et al., 2003). This variability is often dependent on a number of individual or contextual characteristics. Thus, modeling the heterogeneity of the within-person variance and including meaningful covariates to account for why individuals are more variable on some occasions than others is an alternate way to understand process. The present study utilized a 14-day intensive measurement design to examine the effects of fluctuations in daily heart rate on variability in cognitive performance. Fifty-five older adults (Mage = 70.1 years) completed daily cognitive tasks and measures of well-being, while also wearing an accelerometer to capture physical activity, sleep, and continuous heart rate. Data were examined with a mixed effects location scale model (Hedeker et al., 2008; Rast et al., 2012), which incorporates both an individual’s measured level (location) and their variability around that level (scale). Both the location and the scale are permitted to be random such that on any given occasion an individual may deviate from their typical level and they may also deviate from their typical amount of variability. In this way, the location scale model allows for the heterogeneity of the variance in cognition to be modeled and accounted for with other covariates. Results revealed that there were significant amounts of variability in daily heart rate and cognition across 14 days. The impact of heart rate variability on cognitive performance will be discussed.

